# Mid-Infrared Trace Gas Sensor Technology Based on Intracavity Quartz-Enhanced Photoacoustic Spectroscopy

**DOI:** 10.3390/s17030513

**Published:** 2017-03-04

**Authors:** Jacek Wojtas, Aleksander Gluszek, Arkadiusz Hudzikowski, Frank K. Tittel

**Affiliations:** 1 Institute of Optoelectronics, Military University of Technology, 00-908 Warsaw, Poland; 2 Electronics Faculty, Wroclaw University of Science and Technology, 50-370 Wroclaw, Poland; aleksander.gluszek@gmail.com (A.G.); hudzikowski@gmail.com (A.H.); 3 Department of Electrical and Computer Engineering, Rice University, Houston, TX 77005-1892, USA; fkt@rice.edu

**Keywords:** laser absorption spectroscopy, bow-tie cavity, I-QEPAS, intracavity quartz-enhanced photoacoustic spectroscopy, mid-infrared trace gas sensors

## Abstract

The application of compact inexpensive trace gas sensor technology to a mid-infrared nitric oxide (NO) detectoion using intracavity quartz-enhanced photoacoustic spectroscopy (I-QEPAS) is reported. A minimum detection limit of 4.8 ppbv within a 30 ms integration time was demonstrated by using a room-temperature, continuous-wave, distributed-feedback quantum cascade laser (QCL) emitting at 5.263 µm (1900.08 cm^−1^) and a new compact design of a high-finesse bow-tie optical cavity with an integrated resonant quartz tuning fork (QTF). The optimum configuration of the bow-tie cavity was simulated using custom software. Measurements were performed with a wavelength modulation scheme (WM) using a *2f* detection procedure.

## 1. Introduction

Optoelectronic gas sensors based on laser absorption spectroscopy (LAS) can achieve low minimum detection limits (MDL) and high selectivity [1,2]. Techniques for improving LAS detection sensitivity are based on multipass gas cells [3,4] or optical resonators (cavities) [5,6] in order to increase the effective optical path length. LAS can be explained by Lambert-Beer’s law

(1) $$I(\lambda )=I_{0}(\lambda )\exp\left[-\alpha (T,\lambda )L\right],$$

where *I*_0_(*λ*) is the intensity of the incident radiation, *α* denotes the absorption coefficient of the targeted trace gas, *L* is the length of the optical path in the absorbing sample and *T* is the temperature of the sample [7]. When the wavelength of the incident radiation is matched to an absorption line of the sample, the optical path increases and results in a higher difference between *I*_0_(*λ*) and *I*(*λ*).

An improved MDL can be achieved due to an increase in the signal-to-noise ratio. Wavelength modulation (WM) and frequency modulation (FM) are used to achieve this goal. These methods are applicable for tunable diode lasers absorption spectroscopy (TDLAS–WM/FM) due to the spectral tunability of semiconductor diode lasers. Distributed feedback (DFB) diode lasers are particularly useful for this purpose since they emit a single axial mode output. The wavelength of the probing laser is modulated over the absorption line by a sinusoidal signal of frequency. As a result, the light intensity transmitted through the sample TDLAS gas cell and the signal at the detector has a time-dependent form. The signal is detected with a lock-in amplifier. A slow change of the mean laser frequency provides the opportunity to record the first or the second derivative of the absorption spectrum, depending on the demodulation frequency. The advantage of wavelength modulation spectroscopy (WMS) is that it is proportional to the output signal derivative of the targeted absorption line. As a result, harmonic signals reach a maximum value when absorption is the greatest and the odd harmonic signals pass through zero [2]. Therefore, second harmonic (i.e., *2f* detection) measurements are usually performed, while the first or the third harmonics are used to stabilize the operating point of the laser source. Hence, good detection sensitivity and selectivity are the main advantages of TDLAS. WM and FM modulation can also be combined with other methods of ultrasensitive laser absorption spectroscopy, such as cavity ring down spectroscopy (CRDS) or photoacoustic spectroscopy (PAS). Trace gas sensor systems based on PAS are very effective tools for trace gas sensing [8,9,10] and are characterized by a compact, cost-effective and robust architecture. Sensitive microphones are used to detect the conversion of laser light matched to the targeted absorption line in the trace gas sample [11]. The main disadvantage of PAS sensors is their sensitivity to mechanical and acoustic vibrations. A much improved sensor performance can be obtained by replacing the microphone with a commercially available, inexpensive quartz tuning fork (QTF). Such a technique is called quartz-enhanced photoacoustic spectroscopy (QEPAS). QTFs have a resonant frequency of ~32.8 kHz and a Q-factor of ~10^5^ in vacuum and ~10^4^ at 760 Torr [12]. Furthermore, only the symmetric vibration of a QTF is piezoelectrically active, which makes QEPAS immune to environmental acoustic noise, applicable over a wide range of pressures and capable of analyzing trace gas samples as small as ~3 mm^3^ [13].

An I-QEPAS sensor platform was first reported by S. Borri et al. based on a combination of QEPAS and an optical resonator [14]. A selective optical filter was used to focus the optical power between the QTF prongs. I-QEPAS achieved an extremely low MDL, because the QEPAS signal amplitude is directly proportional to the available laser excitation power. The target trace gas was carbon dioxide and a CO_2_ detection sensitivity of 300 ppt for a 4 s integration time was achieved.

In this paper we report the application of the I-QEPAS sensor platform to fast measurements of nitric oxide trace concentrations. Because of the intracavity power enhancement factor obtained by a four-mirror cavity, improved detection sensitivity was achieved, since in QEPAS the sensitivity scales directly with the laser power. NO is a toxic gas, the concentration of which is strongly related to meteorological conditions and emission sources, which can be both natural and anthropogenic. NO is produced during combustion of fossil fuels in power plants and automobile engines as well as during lightning in thunderstorms. Droplets and vapor of nitric acid are removed from the atmosphere in rain or dry precipitation of aerosols [15]. During daytime, there is a correlation between the concentration of NO_x_ and NO_2_, and during nighttime between NO_x_ and NO. Nitric oxide is also a characteristic decomposition compound of specific explosive materials [7]. Furthermore, NO plays an important role in numerous functions in the human body where it is produced in inflammatory processes. In 1998, three US scientists, Robert F. Furchgott, Louis J. Ignarro and Ferid Murad, received the Nobel Prize in Physiology or Medicine for their discoveries concerning the role of "nitric oxide as a signaling molecule in the cardiovascular system" [16]. Thus, real-time concentration measurements of short-lived NO are important in many applications. At present, most common commercially available NO analyzers, which offer concentration measurements at the ppb level, are based on chemiluminescence. Such analyzers detect the UV radiation produced in the reaction of NO with ozone [17,18]. Another approach consists of measuring the change in the ozone concentration instead of the UV intensity [19]. Furthermore, the I-QEPAS–based sensor technology is equally applicable to other trace gas species.

## 2. Experimental Setup

**Figure 1:**
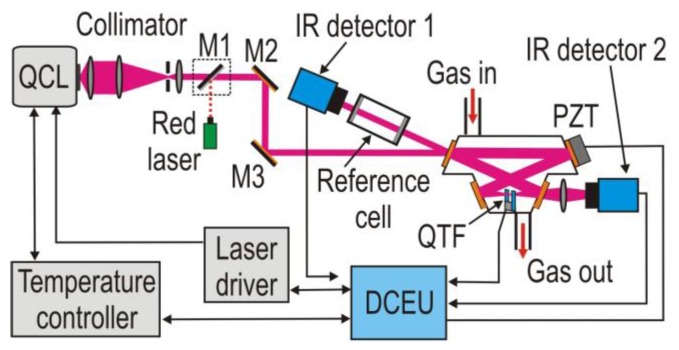
Schematic of an I-QEPAS nitric oxide sensor platform.

NO has a strong absorption band in the mid-infrared spectral range located at a wavelength of 5.263 µm (1900.08 cm^−1^). The pressure inside the I-QEPAS multipass gas cell (MPGC) was reduced in order to minimize the interference from CO_2_ and H_2_O. The measurements were performed with wavelength modulation (WM) using a *2f* detection scheme. The WM technique suppresses background noise originating from spectrally non-selective absorbers (such as the resonator walls, QTF electrodes, and MPGC elements). A diagram of the I-QEPAS sensor platform is shown in Figure 1.

**Figure 2:**
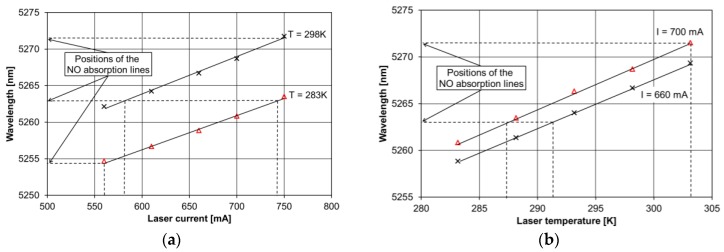
(**a**) Measurements results of the QCL current; and (**b**) the temperature influence on the QCL wavelength.

A room-temperature, continuous-wave (cw), distributed-feedback (DFB) QCL (Hamamatsu Photonics) emitting at 5.263 µm wavelength enclosed in a High Heat Load (HHL) package was used as the excitation source. The linewidth of a DFB QCL is narrow and mainly limited by the noise of the QCL current and temperature controllers [20]. The QCL emission wavelength can be matched to the NO molecular absorption spectrum as shown in Figure 2. Wavelength measurements were carried out using a FT-IR spectrometer (Thermo Fisher Scientific, Waltham, MA, USA). QCL wavelength tuning was demonstrated from 5.255 µm (using a laser current of 560 mA at a laser temperature of 283 K) to 5.272 µm spectral range (using a laser current of 700 mA at a laser temperature of 298 K).

**Figure 3:**
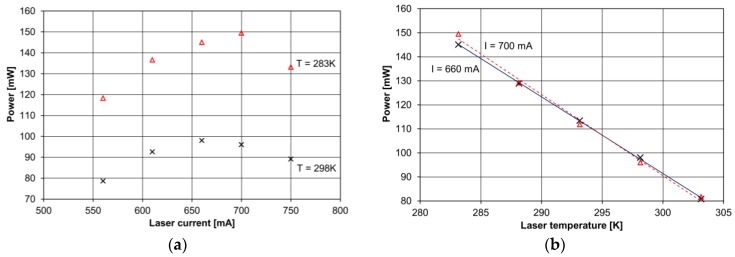
(**a**) Measurements results of laser current; and (**b**) temperature influence on optical power of QCL radiation.

The QCL radiation was collimated by a 5.95 mm focal length black diamond aspheric lens (type 390028-E, Thorlabs, Newton, NJ, USA). In addition a collimator consisting two ZnSe lenses and a 300 µm pinhole were applied. Such an optical setup resulted in a high power mid-infrared excitation beam (150 mW @ 700 mA and 283 K) with a diameter of 1.7 mm and a divergence <1 mrad. Figure 3 depicts the available QCL excitation power measured at the collimator output. The QCL power measurements were performed for different QCL currents and temperatures using a power meter (OPHIR-Nova II, Ophir Optronics Solutions Ltd., Jerusalem, Israel). A red diode alignment laser and a movable mirror M1 were used for optimum alignment of the mid-infrared I-QEPAS optical system.

The QCL beam from the collimator was directed by means of two gold coated plane mirrors (M2 and M3) to the compact, high-finesse bow-tie optical cavity. The 12 cm^3^ bow-tie optical cavity was formed by four half-inch-diameter (two plane and two concave mirrors with a 30 mm radius of curvature), highly reflective dielectric mirrors (R > 99.99% from LohnStar Optics, Escondido, CA, USA). The use of such mirrors resulted in a cavity finesse of 7854 (F = π/l_r_, where l_r_ denotes the losses per round trip due to mirrors transmission). Such an optical resonator design resulted in a power built-up inside the cavity of 2500 times (G = F/π) [21].

A commercially available quartz tuning fork (QTF) equipped with micro-resonator was applied in our I-QEPAS setup [18]. The QCL beam must pass through the gap between the QTF prongs in order to achieve efficient excitation of the QTF’s symmetric vibration. The measurements were performed using a lock-in amplifier and a WM-*2f* detection scheme that suppresses the background originating from spectrally non-selective absorbers such as the resonator walls, the QTF electrodes and the gas cell elements. A reference cell filled with a high concentration of the targeted gas and an IR detector-1 were used to register the *1f* or *3f* signals. Further details related to experimental setup are reported in Section 4.

## 3. Cavity Design

**Figure 4:**
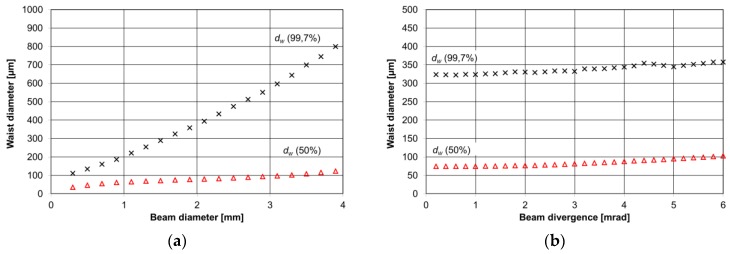
(**a**) Simulation results of beam diameter; and (**b**) divergence of a 4 mm beam influence on a waist diameter *d_w_* inside a four mirror bow-tie cavity.

**Figure 5:**
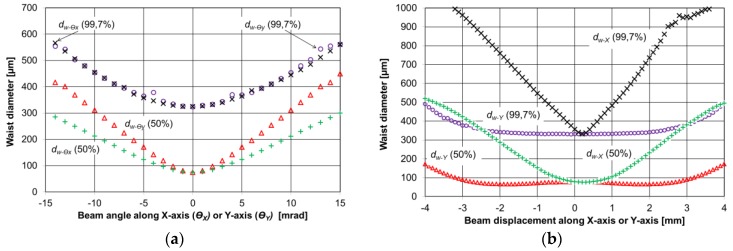
(**a**) Results of simulation of the QCL 4 mm beam waist diameter (*d_w_*) inside the cavity versus the QCL beam angle; and (**b**) the beam displacement.

The dimensions and mirror positions of the cavity were specified and designed in Autodesk Inventor software and simulated by means of custom software. The distance between the mirrors was slightly increased (~0.5 mm more than the mirror focal point) to achieve a stable, focused beam in the bow-tie cavity based on our simulations. The modeling of the QCL beam reflection on a spherical mirror and the intersection point between a light ray and the mirror sphere was determined first. The intersection points were found with equations reported in Reference [22]. The direction of the reflected beam was calculated based on the law of reflection. Performing a Gaussian laser beam analysis required the generation of a group of ideal rays. Each ray has a different origin of coordinates and different angles (directions) corresponding to the beam size and divergence. Rays are reflected at each mirror ~10^3^ times, taking into account associated power losses. Round-off errors were negligible when using 64-bit floating point variables. The QCL beam power at the focal point (waist diameter—*d_w_*) was determined by the recorded sum of each ray power at the intersection of a plane located at the focal point. Two QCL beam sizes at the focal point were calculated, a first beam size with 50% of the total power and a second beam size with 99.7% (3σ) of the total power. Simulations were performed for various QCL beam X-Y positions, divergence angles and beam sizes. The results of the calculation related to cavity performance are presented in Figure 4 and Figure 5 (astigmatic distortions were neglected).

Figure 4 shows that the bow-tie cavity can provide a small waist diameter (<1 mm) at the QTF position, even for a QCL beam with a 4 mm diameter, and is stable for varying beam divergences. The bow-tie cavity design is immune to QCL beam displacement. The angles Ɵ_x_ and Ɵ_y_ should be close to zero in order to achieve the smallest beam waist (Figure 5). For Ɵ_y_, even a 4 mrad angle leads to a two-times-bigger beam waist diameter for 50% of the total power. The change of the beam position along the X-axis is more important than changes along the Y-axis, where the QCL beam can be moved up to 3 mm in both directions without significant beam waist changes.

## 4. Experimental Results

**Figure 6:**
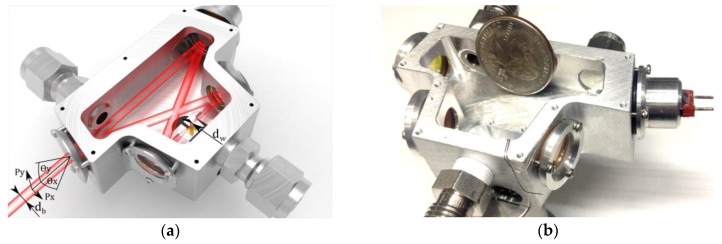
(**a**) Optical cavity 3D design; and (**b**) photo of a novel and compact bow-tie resonator for an I-QEPAS sensor system.

The bow-tie enclosure was fabricated from an aluminum block with the shape determined by the calculated positions of the mirrors (Figure 6). The angle between the mirrors was <12° and the outside dimensions were 68 × 46 × 25 mm. The internal and external surfaces of the cavity were the base for all the optical, mechanical and electrical components needed to assemble a complete sensor system consisting of mirrors, the QTF and a piezoelectric transducer (PZT). The mirrors could be aligned precisely by three-point kinematic mounts with metal O-rings and silicone sealing. The QTF could be aligned by means of a sliding mount pressed into the bow-tie enclosure by two springs. This method makes it possible to align the QTF before locking its position by means of epoxy.

Interference from the cavity mirror feedback can be eliminated, because the QCL beam can be injected at a small angle with respect to the input mirror axis. Hence, a costly optical isolator was not required. In the beam reflected by the input mirror, a reference gas cell filled with pure NO (3 cm in length and a 0.85 cm diameter) and a mercury cadmium-telluride (MCT) detector-1 (PVI-4TE, VIGO System S.A., Ozarow Mazowiecki, Poland) were placed for QCL wavelength monitoring.

The total cavity length was L = 193 mm, corresponding to a free spectral range (FSR = c/L) of 1.55 GHz. One of the plane mirrors was mounted on a PZT and provided precise tuning of the cavity length in the range of 11 µm along the z-axis to achieve mode matching with the QCL radiation. The intracavity optical power was determined to be ~170 W by measuring the optical power reflected from the input cavity mirror. Hence, a power enhancement factor of 1276 was achieved with respect to the incident QCL power of 133.2 mW achieved with a QCL drive current of 742.5 mA and an operating temperature of 283 K.

**Figure 7:**
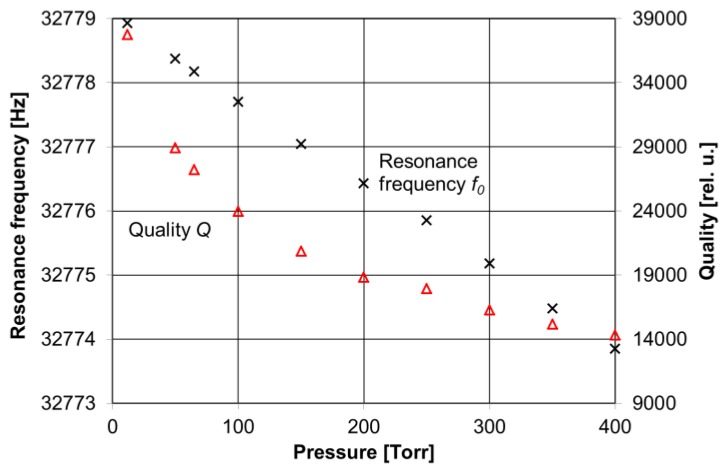
QTF resonance frequency and quality factor measurements as a function of pressure inside the optical cavity.

The cavity was designed to focus the laser beam between the two spherical mirrors. At the focal plane of these two mirrors, the QTF was mounted on a custom-machined miniature xyz translator. The QCL beam passed through a 0.5 mm gap between the QTF prongs without hitting them. The electrical parameters of the QTF (i.e., the dynamical resistance, the quality factor *Q* and the resonant frequency *f_0_*) were measured with a function generator (DS345, Stanford Research System), a lock-in amplifier (SRS830, Stanford Research System) and LabVIEW software. The measured QTF resonance frequency was *f*_0_ = 32,776.2 Hz and the quality factor *Q* exceeded 28,900 (see Figure 7).

**Figure 8:**
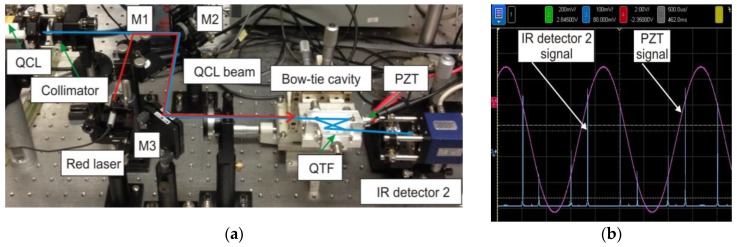
(**a**) Photos of the experimental setup; and (**b**) an example of recorded PZT and I-QEPAS signals.

The experimental setup is depicted in Figure 8. A MCT detection module (PVI-4TE, VIGO System S.A.) detector-2 was used to measure the exiting QCL radiation. A custom digital control electronic unit (DCEU) based on a STM32F7 microcontroller platform equipped with an analog front-end system was developed to generate all the signals required by the I-QEPAS sensor system. The modulation of the QCL current was performed by a low-amplitude sinusoidal signal with a frequency corresponding to half of the QTF resonance frequency, *f*_0_/2 (~16.4 kHz). The output signal from the QTF was connected to a transimpedance preamplifier and subsequently directed to a custom lock-in amplifier detecting the second harmonic of the QTF signal (~32.8 kHz).

The PZT transducer shown in Figure 1 and Figure 6 was controlled by a digital high-speed feedback loop using the signal from the IR detector-2. This signal tracked the amplitude of the I-QEPAS signal in order to achieve the highest power inside the four-mirror optical resonator. The PZT driving signal and output detector-2 signal are shown in Figure 8b. Thin lines registered by detector-2 are the result of the high finesse of the cavity.

The I-QEPAS–based NO concentration measurements were carried out in a locked mode. The QCL frequency was initially set to the center of the absorption line and the signal strength was monitored by the IR detector-2. A custom algorithm based on a PID (Proportional, Integral, Derivative algorithm) with conditional executing and phase wrapping functions provided the signal to control the PZT driver. Such a feedback loop maintained a high QCL power inside the bow-tie cavity.

**Figure 9:**
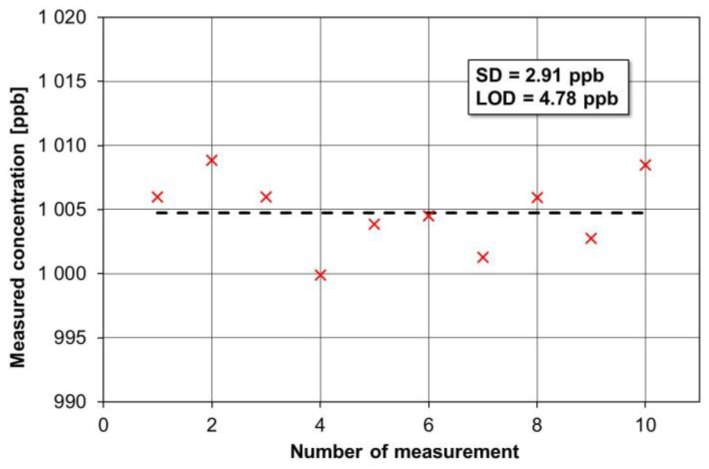
Results of NO concentration measurements.

The absorption line at 1900.08 cm^−1^ was found to be optimal for NO detection, as already mentioned in Section 2. An NO absorption cross-section of 0.7 × 10^−18^ cm^2^ was observed at this wavenumber. A detailed description of NO absorption line selection can be found in Reference [23]. Measurements of a 1 ppm NO sample at 50 Torr were carried out. Both the gas pressure and the wavelength modulation depth to optimize the NO I-QEPAS detection performance were determined experimentally. The optimal amplitude of the modulation signal for scanning of the cavity mode (FWHM ~0.2 MHz) was obtained by measuring the I-QEPAS signal as a function of the QCL modulation current at the optimum pressure inside the MPGC. A critical task was to minimize the noise of the QCL controller using low-noise custom electronics because of the narrow cavity mode FWHM. To determine the MDL, defined as the lowest amount of trace gas that can be distinguished from the system noise, we used a signal-to-noise ratio (SNR) and a 90% confidence level that corresponds to a 1.645 SD (standard deviation). Figure 9 shows the NO concentration measurements results. The sensor signal was measured with a 1.01 ppm NO of a high-purity calibration mixture and the noise as fluctuations of this concentration level. The measurements were carried out with a 30 ms integration time. A difference smaller than 0.5% between the average value of the measurements and the nominal concentration of the calibration mixture was measured.

The MDL of the I-QEPAS NO sensor operating in a locked mode at λ ~5.263 μm was ~4.8 ppbv with a 30 ms integration time due to the cavity stability and effective locking between the QCL and the bow-tie cavity. The MDL was limited by distortions of the PZT driving signal and its adverse influence on the NO concentration measurements. This effect occurred because the PZT required a high-amplitude voltage signal (up to 10 V) and its harmonics were located near the output signal frequency.

## 5. Conclusions

In conclusion, the first demonstration of intracavity quartz-enhanced photoacoustic spectroscopy applied to mid-infrared NO detection using a compact bow-tie cavity configuration with a minimum detection limit of ~4.8 ppbv was reported. The bow-tie cavity provided a high finesse and a small waist diameter (<1 mm) as well as a minimum beam divergence and beam displacement. Low-noise digital electronics were developed to ensure the effective coupling of the CW, DFB QCL emission line to a narrow bow-tie cavity mode. Further improvements of minimizing the influence of the PZT noise can improve the MDL by approximately one order of magnitude.

An I-QEPAS–based sensor is a novel ultra-sensitive and selective platform for trace gas sensing and is useful in many applications including environmental monitoring, industrial emission measurements, chemical analysis, security and medical diagnostics [24] as well as in the life sciences [1,25]. NO plays a very important role in atmospheric chemistry as it controls the ozone concentration and production rates in the stratosphere by the photodissociation process [15]. Other trace gases detectable by I-QEPAS include NO_2_, N_2_O, HNO_3_, CH_2_O, SO_2_, O_3_, CO and NH_3_. Furthermore, I-QEPAS sensors are ultra-sensitive, compact and less costly than techniques that require costly photodetectors.
